# Association of TLR4 and Treg in *Helicobacter pylori* Colonization and Inflammation in Mice

**DOI:** 10.1371/journal.pone.0149629

**Published:** 2016-02-22

**Authors:** Yanfeng Gong, Liming Tao, Lei Jing, Dongsheng Liu, Sijun Hu, Wei Liu, Nanjin Zhou, Yong Xie

**Affiliations:** 1 Department of Gastroenterology, The First Affiliated Hospital of Nanchang University, No. 17, Yongwai Zheng Street, Nanchang, Jiangxi, China; 2 Department of Geriatrics, The First Affiliated Hospital of Nanchang University, No. 17, Yongwai Zheng Street, Nanchang, Jiangxi, China; 3 Department of Biochemistry and Molecular Biology, Jiangxi Academy of Medical Science, Jiangxi, China; 4 Department of Obstetrics, The First Affiliated Hospital of Nanchang University, No. 17, Yongwai Zheng Street, Nanchang, Jiangxi, China; Oita University Faculty of Medicine, JAPAN

## Abstract

The host immune response plays an important role in the pathogenesis of *Helicobacter pylori* infection. The aim of this study was to clarify the immune pathogenic mechanism of *Helicobacter pylori* infection via TLR signaling and gastric mucosal Treg cells in mice. To discover the underlying mechanism, we selectively blocked the TLR signaling pathway and subpopulations of regulatory T cells in the gastric mucosa of mice, and examined the consequences on *H*. *pylori* infection and inflammatory response as measured by MyD88, NF-κB p65, and Foxp3 protein expression levels and the levels of Th1, Th17 and Th2 cytokines in the gastric mucosa. We determined that blocking TLR4 signaling in *H*. *pylori* infected mice decreased the numbers of Th1 and Th17 Treg cells compared to controls (P < 0.001–0.05), depressed the immune response as measured by inflammatory grade (P < 0.05), and enhanced *H*. *pylori* colonization (P < 0.05). In contrast, blocking CD25 had the opposite effects, wherein the Th1 and Th17 cell numbers were increased (P < 0.001–0.05), immune response was enhanced (P < 0.05), and *H*. *pylori* colonization was inhibited (P < 0.05) compared to the non-blocked group. In both blocked groups, the Th2 cytokine IL-4 remained unchanged, although IL-10 in the CD25 blocked group was significantly decreased (P < 0.05). Furthermore, MyD88, NF-κB p65, and Foxp3 in the non-blocked group were significantly lower than those in the TLR4 blocked group (P < 0.05), but significantly higher than those of the CD25 blocked group (P < 0.05). Together, these results suggest that there might be an interaction between TLR signaling and Treg cells that is important for limiting *H*. *pylori* colonization and suppressing the inflammatory response of infected mice.

## Introduction

*Helicobacter pylori* (*H*. *pylori*) are Gram-negative spirobacteria that colonize gastric epithelial cells as well as the gastric mucosa. Although most infected individuals are asymptomatic, *H*. *pylori* infection has been linked to chronic gastritis, peptic ulcer diseases, and lymphomas of the gastric mucosa-associated lymphoid tissue [1]. The World Health Organization has categorized *H*. *pylori* as a class I carcinogen/definite human carcinogen. Understanding the mechanism of *H*. *pylori* infection could play an important role in its treatment. Presently, however, there have been few studies on this topic, and the mechanism remains unclear.

Some studies have found that Toll-like receptors (TLRs) and regulatory T cells (Treg) play roles in the immune pathogenesis of *H*. *pylori* infection. TLRs, which are transmembrane proteins that act mainly as sensors of microbial components, could recognize the unique pathogen-associated molecular patterns of *H*. *pylori*. TLRs participate in detecting pathogens, are an important component of the innate immune response [2], and play a pivotal role in the induction of both innate and adaptive immune responses during immune defense. *H*. *pylori* intimately contact gastric epithelial cells during infection; these cells play an important role in signal transduction of the innate immunity induced by *H*. *pylori*. TLRs such as TLR2, TLR4, TLR5, and TLR9 have been found to be widely expressed on gastric epithelial cells [3]. Some research has shown that infection by *H*. *pylori* and the particular *H*. *pylori* composition thereof can influence TLR up-regulation, and that TLRs in turn had an important role in recognizing *H*. *pylori* and stimulating the immune and inflammatory response induced by infection [4]. For example, Wang et al. [5] detected TLR4 in the gastric epithelium and on monocytes/macrophages in the atrophy and intestinal metaplasia of superficial gastritis, and found that the number of TLR4 positive cells in these tissues was much larger in the presence of *H*. *pylori* infection than in uninfected tissues, and that TLR4 expression was significantly correlated with the severity of inflammation. Li et al. [6], in an analysis of TLR4 polymorphisms within Chinese patients with gastric cancer and atrophic gastritis, found that the TLR4-2081G/A polymorphism seemed to affect the risk of gastric carcinogenesis and to some degree played a protective role against *H*. *pylori* infection. Kawahara et al. [7] found that *H*. *pylori* and lipopolysaccharide (LPS) could stimulate the expression of TLR4 on gastric pit cells of the guinea pig, and induce inflammatory response therein. In contrast, another study found that the LPS of some *H*. *pylori* strains could antagonize TLR4 [8]. Therefore, the response of TLR4 on gastric epithelial cells to *H*. *pylori* LPS was very low, which might be one strategy by which *H*. *pylori* escapes host immunity defense mechanisms. Together, these research results indicated that TLRs were both the activating factor underlying *H*. *pylori* induction of host immunity and an important mechanism by which *H*. *pylori* could escape host immunity defenses.

Tregs, which are a T cell population having regulatory functions, differ from Th1 and Th2 effector T cells, and have been shown to suppress immune response [9]. Among these, CD4^+^CD25^+^ Tregs are the most important. Recent studies have shown that these represent the key mechanism of immunity suppression, and could secrete several suppressive cytokines such as IL-4, IL-10, and TGF-β to suppress immunity response. CD4^+^CD25^+^ Tregs have also been shown to induce immune tolerance through suppression of the host immunity response to self or non-self antigens [10]. Some experiments have confirmed that removing or reducing CD4^+^CD25^+^ Tregs could enhance the host anti-infection immunity to several pathogens. In other words, endogenous CD4^+^CD25^+^ Tregs were able to suppress the effective protection immunity response of the host to invading pathogens, resulting in long-lasting pathogenic infection [11]. Furthermore, some studies have shown that Treg play a role in the ability of *H*. *pylori* to escape host immunity elimination. Lundgren et al. [12] found that the levels of CD4^+^CD25^+^ Tregs in the gastric and duodenum mucosa of humans infected with *H*. *pylori* was higher than those in non-infected mucosa, and that this could suppress the gastric mucosal immune response to *H*. *pylori*. Raghavan et al. [13] respectively infused either lymphonodus cells or lymphonodus cells from which the Tregs had been removed into athymic mice, which were then infected with *H*. *pylori*. They found that the *H*. *pylori* colonization density of the latter was significantly lower than that of the former. These studies indicated that Tregs might be another strategy by which *H*. *pylori* escapes elimination by host immune response mechanisms.

To date, an increasing number of studies have indicated that the TLR signaling pathway might interact with Tregs. The TLR signaling pathway could facilitate resistance to the immune suppression induced by Tregs through producing cytokines such as IL-6 and IL-1 [14]. Cao et al. [15] found that Foxp3^+^ Tregs were increased in the intestines of TLR4- or IL-10-blocked mice, and that TLR4 signaling inhibited Foxp3^+^ induction. Lacave-Lapalun et al. [16] indicated that after colorectal irradiation, treatment with LPS, a TLR4 ligand, resulted in overexpression of Foxp3. TLRs could also regulate Treg function through a MyD88-dependent pathway [14,17]. Voo et al. [18] used a human peripheral blood mononuclear cell-based proliferation assay system to simultaneously monitor the effects of TLRs on T cells, and found that TLRs could block Treg suppression of CD4^+^ or CD8^+^ T cell proliferation. In addition, Tregs might also affect TLR function. Murphy et al. [17] found that infusing Tregs to mice with burn wounds could reduce the cytokine production stimulated by TLRs. Furthermore, Okeke et al. [19] showed that injection of LPS led to expansion of CD4^+^CD25^+^Foxp3^+^ Tregs and inhibition of Treg function in mice lacking functional Tregs (CD25 knockout mice), and that application of an anti-CD25 monoclonal antibody led to acute death in an otherwise nonlethal LPS challenge. Wang et al. [20] also found that the expression of CD4^+^CD25^+^ Tregs was reduced, and Foxp3 was down-regulated, but the expression levels of TLRs were up-regulated in patients with infectious mononucleosis at the acute stage. Overall, these findings suggest that there exists an interaction between TLRs and Tregs such that when TLRs are blocked, the expression and function of Tregs increase, and when Tregs are suppressed, the expression of TLRs become augmented.

In conclusion, current research suggests that the TLR signaling pathway can interact with Treg, and that *H*. *pylori* infection would have an effect on both TLR4 and Treg signaling pathways. However, it was not very clear whether such an interaction exists during *H*. *pylori* infection, and if so, whether it would affect *H*. *pylori* colonization and inflammation. In 2014, Kabisch et al. [21] used TLR4 neutralization dendritic cells co-incubated with *H*. *pylori*, and found that the consequent expression of Foxp3 was reduced. As TLR4 and Tregs were only examined *in vitro*, the effect on the interaction between the TLR4 and Treg signaling pathways with *H*. *pylori* colonization and inflammation was unclear. To explore this issue, we respectively blocked TLR4 and CD25 in mice, and studied the effects of the TLR signaling pathway and of Treg individually and together on *H*. *pylori* colonization and inflammation, to illustrate the immune protective mechanism by which *H*. *pylori* escapes host immunity elimination. Our findings might provide a new strategy for designing effective preventive and therapeutic treatment regimens against *H*. *pylori*.

## Materials and Methods

### Reagents and bacterial strains

Chitosan and 88.5% deacetylated chitosan powder were purchased from Qisheng Biological Products Limited Company, Shanghai, China. Anti-Fc, anti-TLR4, and anti-CD25 monoclonal antibodies were purchased from eBioScience, San Diego, CA, USA. ELISA kits for IFN, IL-12, IL-17, IL-4, and IL-10 were purchased from eBioScience. Goat-anti-mouse Foxp3, rabbit-anti-rat NF-κB p65, and rabbit-anti-rat MyD88 polyclonal antibodies were purchased from Abcam, Cambridge, UK. A Molecular Dynamics (Sunnyvale, CA, USA) densitometer and ImageQuant 5.0 software (Amersham Biosciences, Piscataway, NJ, USA) were used. The BH-2 stereo-binocular microscope was purchased from Suzhou REIT Image Technology Co Ltd, Suzhou, China. *H*. *pylori* Sydney strain 1 (SS1) was kindly provided by the *H*. *pylori* Strain Pool, Beijing, China.

### Animal care and use statement

Female BALB/c mice, 6–8 weeks of age and 22.5 g of mean weight, were purchased from Vitalriver Experimental Animal Technology Co. Ltd., Beijing, China. The mice were housed in a specific pathogen-free environment with free access to food and water. All animal experiments were conducted in accordance with principles stated in the Guide for the Care and Use of Laboratory Animals (NIH publication 8623, National Institutes of Health, Bethesda, MD, 1985). These experimental protocols were approved by the Ethics Committee of the First Affiliated Hospital of Nanchang University.

### Culture of *H*. *pylori*

*H*. *pylori* SS1 were used throughout the experiments. *H*. *pylori* was grown in *Campylobacter* agar base containing 10% sheep blood under microaerobic conditions (5% O_2_, 10% CO_2_, and 85% N_2_) at 37°C for 2–3 days.

### Groups

BALB/c mice were randomly divided into six groups: 1) Control group (10 mice); 2) TLR4 blocked control group (10 mice); 3) CD25 blocked control group (10 mice); 4) *H*. *pylori* infection group (10 mice); 5) TLR4 blocked *H*. *pylori* infection group (9 mice); and 6) CD25 blocked *H*. *pylori* infection group (10 mice).

### TLR4 and Treg signaling pathway block

At 4–5 days prior to *H*. *pylori* infection, subject mice in the CD25 blocked group were given Fc antibody (100 μg/mouse) by intraperitoneal injection. Subsequently, CD25 antibody (1.2 mg/kg weight) was intraperitoneally injected[22]. The TLR4 blocked group received intraperitoneal injection of the TLR4 antibody (20 μg/mouse) 1 day after intraperitoneal injection of the Fc antibody (100 μg/mouse). The non-blocked group was intraperitoneally injected with rat IgG1 (20 μg/mouse)[23].

### *H*. *pylori* infection

Each mouse was orally administered 1×10^9^ colony-forming units (CFUs) *H*. *pylori* per L five times every other day. At 12 weeks after the last inoculation, four mice were euthanized, and the stomachs were removed to confirm that the *H*. *pylori* infection model was established. Subsequently, mice were all euthanized by inhalation ether, and gastric tissue was processed for further analyses as decribed later.

### Assessment of bacterial load in the stomach

The bacterial load in the stomach was determined by Giemsa staining. *H*. *pylori-*negative was defined when Giemsa staining was determined to be negative by visual observation, and *H*. *pylori*-positive was defined when positive Giemsa staining was observed. The colonization was assessed by semiquantitative analysis of *H*. *pylori* levels in the gastric mucosa (0 cells/crypt = 0; 1–2 cells/crypt = 1; 3–10 cells/crypt = 2; 11–20 cells/crypt = 3; > 21 cells/crypt = 4).

### Assessment of gastritis in the stomach

The levels of gastritis in the stomach were determined by hematoxylin and eosin (HE) staining. The grades of gastritis were assessed by semiquantitative analysis of chronic inflammatory cells such as lymphocytes and plasmocytes in the lamina propria of the gastric mucosa (few lymphocytes = 0; sporadic lymphocytes and plasmocytes = 1; moderate lymphocytes and plasmocytes = 2; many lymphocytes and plasmocytes = 3).

### Determination of MyD88, Foxp3, and NF-κB p65 protein expression in the gastric mucosa by immunohistochemistry

Snap-frozen biopsies were cut into 4 μm sections to determine the MyD88, NF-κB p65, and Foxp3 protein expression levels in the gastric mucosa by immunohistochemistry. The sections were dewaxed according to standard protocol, sealed by 3% H_2_O_2_ for 8–10 min, treated with microwave radiation for 10 min, and incubated with the primary antibody at 4°C overnight. For detection, the sections were incubated with streptavidin-peroxidase for 10 min at room temperature, then were dyed with DAB solution as the substrate. To exclude nonspecific staining in each case a negative control was performed. For this purpose polyclonal TLR4 and Foxp3 antibodies were blocked with the peptide used for antibody production at 37°C for 20 min. In MyD88-stained sections, positive cells were assigned as those with dyed cell membranes or yellow to brown-dyed cytoplasm, and negative cells were assigned as those with nondyed cell membranes or cytoplasm. In sections stained with either NF-κB p65 or Foxp3, positive cells were assigned as those with the cell nucleus dyed yellow to brown, and negative cells were assigned as those with a nondyed nucleus. The immunoreaction was graded according to the depth of color and the proportion of positive cells. The degree of dyeing was divided into the following score grades: 0 (negative), 1 (yellow, weakly positive), 2 (light brown, positive), and 3 (brown, strongly positive). The fraction of dyeing was expressed as a percentage.

### Determination of MyD88 and Foxp3 protein expression in the gastric mucosa by western blotting

The gastric mucosa was lysed in lysis buffer solution. The lysate was immunologically tested after SDS-polyacrylamide gel electrophoresis (SDS-PAGE). The experimental conditions were as follows (Table 1). The optical density value of the objective band was read from the X-ray film using ImageQuant 5.0 software. MyD88 and Foxp3 protein expression was standardized against the value of the β-actin band in a corresponding group.

**Table 1:** Western blot detection conditions for MyD88 and Foxp3.

Target protein	Molecular weight (kD)	Sealing time and temperature	Primary antibody dilution	Primary antibody incubation time	Secondary antibody dilution	Secondary antibody incubation time
MyD88	35	2 h, room temperature	1:1000	overnight	1:100000	4 h
Foxp3	47	30 min, 4°C	1:2000	overnight	1:100000	4 h

### Determination of cytokines in the gastric mucosa by ELISA

After being weighed, the gastric mucosa was homogenized in 1.3 ml PBS and the homogenates were centrifuged at 3000 × *g* at 4°C for 20 min. ELISA kits were used to quantify IFN, IL-12, IL-17, IL-4, and IL-10 in the supernatants (diluted at 1:2) after centrifugation. The limit of detection was 3 pg/ml for IL-4, 22 pg/ml for IL-10, 6 pg/ml for IL-12, 8 pg/ml for IL-2, and 8 pg/ml for IFN. The results were represented as pg/mg wet weight of the gastric mucosa.

### Statistical analysis

The measurement data were expressed by means ± standard deviation. Differences in multiple groups were analyzed by analysis of variance, and differences between two groups were analyzed by an F-q test (ANOVA). Ranked data were analyzed by the Kruskal-Wallis H test. SPSS 11.5 (SPSS, Chicago, IL, USA) was used for analysis. A *P*-value < 0.05 was considered statistically significant.

## Results

### *H*. *pylori* colonization

To determine whether differences in *H*. *pylori* infection in the gastric mucosa occurred following TLR4 or CD 25 blockage after infection, we measured the *H*. *pylori* colonization scores following pathological tests of the gastric mucosa.

**Fig 1:**
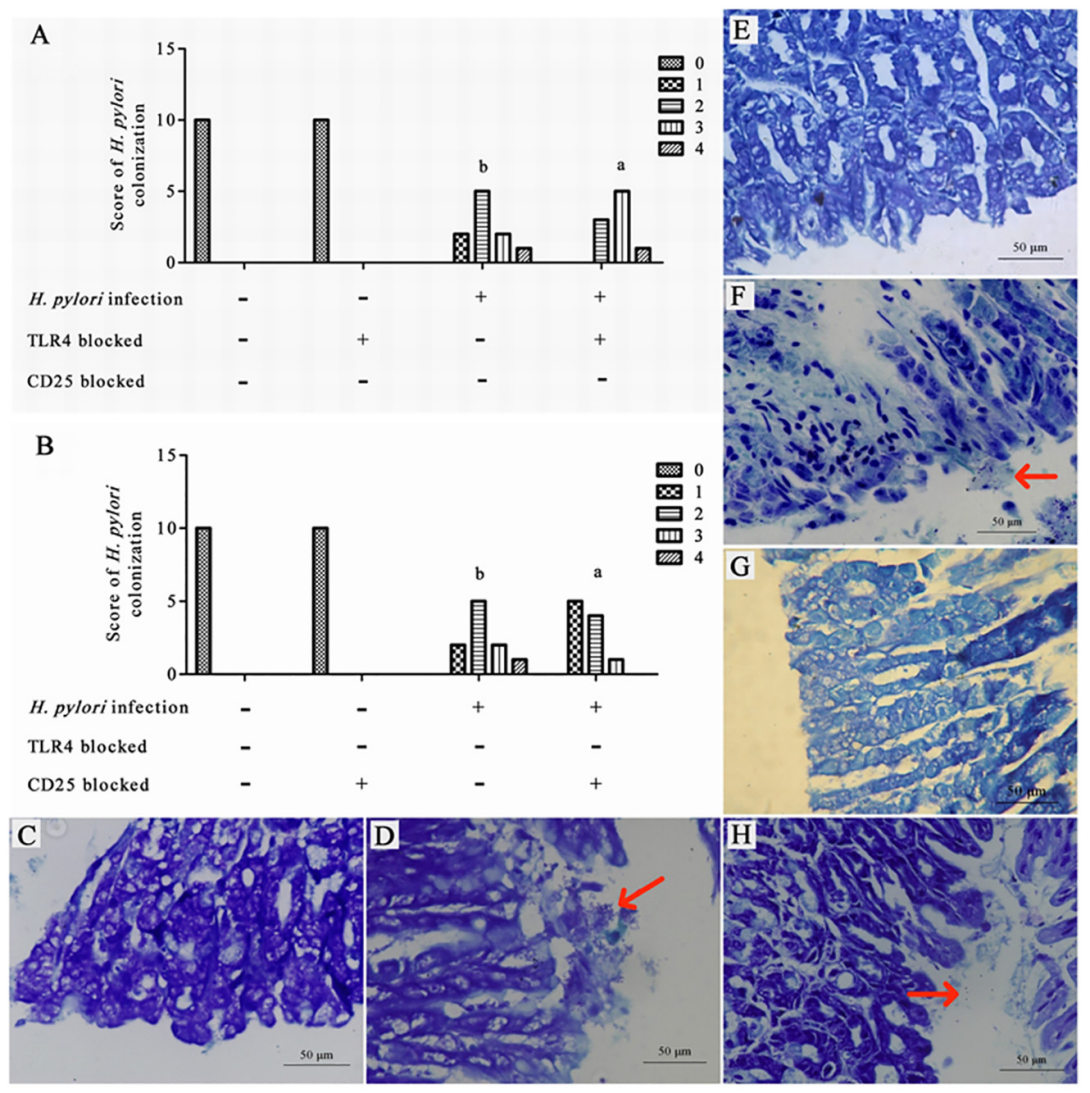
*H*. *pylori* colonization score in the gastric mucosa after infection. (A) *H*. *pylori* colonization score in the gastric mucosa with TLR4 blocked; (B) *H*. *pylori* colonization score in the gastric mucosa with CD25 blocked; (C) Giemsa staining of the gastric mucosa of the control group; (D) Giemsa staining of the gastric mucosa of the *H*. *pylori* infection group; (E) Giemsa staining of the gastric mucosa of the TLR4 blocked control group; (F) Giemsa staining of the gastric mucosa of the TLR4 blocked *H*. *pylori* infection group; (G) Giemsa staining of the gastric mucosa of the CD25 blocked control group; (H) Giemsa staining of the gastric mucosa of the CD25 blocked *H*. *pylori* infection group. ^a^*P* < 0.001 between *H*. *pylori* and control or TLR4 blocked control groups; ^b^*P*< 0.05 between *H*. *pylori* and TLR4 blocked *H*. *pylori* groups (Fig 1A). ^a^*P* < 0.001 *vs*. control or CD25 blocked control groups; ^b^*P* < 0.05 between *H*. *pylori* and CD25 blocked *H*. *pylori* groups (Fig 1B).

After *H*. *pylori* infection, the *H*. *pylori* colonization density was significantly different among the different groups (P = 0.000), and was not significantly different between the control group in which TLR4 was blocked and the control group in which TL4 was not blocked (P>0.05). In the TLR4-blocked H. pylori infection group, the *H*. *pylori* colonization density was significantly higher than that in the control group, regardless of whether or not TLR4 was blocked (P<0.001). The *H*. *pylori* colonization density in the non-blocked H. pylori infection group was significantly lower than that in the TLR4-blocked H. pylori infection group (P<0.05) (Fig 1A). The *H*. *pylori* colonization density was significantly different among different groups (P = 0.000), and was not significantly different between the control group in which CD25 was blocked and the control group in which CD25 was not blocked (P>0.05). In the H. pylori infection group, regardless of whether or not CD25 was blocked, the *H*. *pylori* colonization density was significantly higher than that in the CD25 blocked and non-blocked control groups (P<0.001). The *H*. *pylori* colonization density in the CD25-blocked H. pylori infection group was significantly lower than that in the non-blocked H. pylori infection group (P<0.05) (Fig 1B). The gastric mucosae of *H*. *pylori*-infected mice were tested for evidence of *H*. *pylori*. Giemsa staining of the gastric mucosa of the control group with or without TLR4 or CD25 blockage showed no *H*. *pylori* colonization in the gastric pits (Fig 1C, 1E and 1G). Giemsa staining of the gastric mucosa of the *H*. *pylori* infection group showed less *H*. *pylori* colonization in the gastric pits than did the TLR4 blocked *H*. *pylori* infection group, and more than could be observed in the CD25 blocked *H*. *pylori* infection group (Fig 1D, 1F and 1H, ×200). (data in S1 and S2 Tables).

### Grades of gastritis

To determine *H*. *pylori* infection generated gastritis in the gastric mucosa with TLR4 or CD25 blockage following infection, we measured the grades of gastritis in the gastric mucosa.

**Fig 2:**
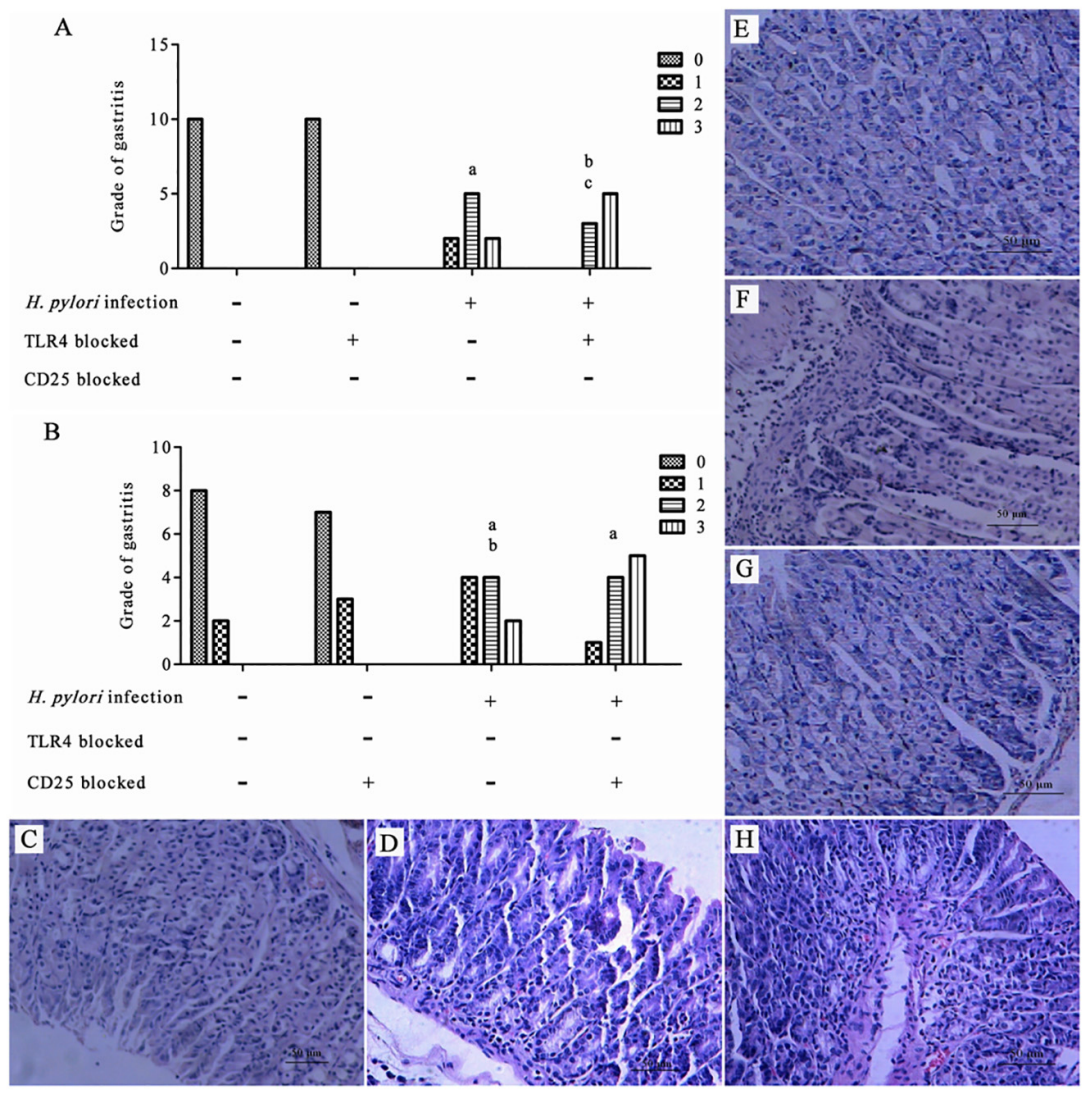
Grade of gastritis after *H*. *pylori* infection. (A) The grade of gastritis with TLR4 blocked; (B) The grade of gastritis with CD25 blocked; (C) HE staining of the gastric mucosa of the control group; (D) HE staining of the gastric mucosa of the *H*. *pylori* infection group; (E) HE staining of the gastric mucosa of the TLR4 blocked control group; (F) HE staining of the gastric mucosa of the TLR4 blocked *H*. *pylori* infection group; (G) HE staining of the gastric mucosa of the CD25 blocked control group; (H) HE staining of the gastric mucosa of the CD25 blocked *H*. *pylori* infection group. ^a^*P* < 0.001 between *H*. *pylori* and control or TLR4 blocked control groups; ^b^*P* < 0.01 between TLR4 blocked *H*. *pylori* and control or TLR4 blocked control groups; ^c^*P* < 0.05 between *H*. *pylori* and TLR4 blocked *H*. *pylori* groups (Fig 2A). ^a^*P* < 0.001 *vs*. control and CD25 blocked control groups; ^b^*P* < 0.05 between *H*. *pylori* and CD25 blocked *H*. *pylori* groups (Fig 2B).

After *H*. *pylori* infection, the grade of gastritis was significantly different among the different groups (P = 0.000), and was not significantly different between the control group in which TLR4 was blocked and the control group in which TL4 was not blocked (P>0.05). In the TLR4-blocked H. pylori infection group, the grade of gastritis was significantly higher than that in the control group regardless of whether or not TLR4 was blocked (P<0.05–0.001). The grade of gastritis in the TLR4-blocked H. pylori infection group was significantly lower than that in the non-blocked H. pylori infection group (P<0.05) (Fig 2A). The grade of gastritis was significantly different among the different groups (P = 0.000), and was not significantly different between the control group in which CD25 was blocked and the control group in which CD25 was not blocked (P>0.05). In the H. pylori infection group, regardless of whether or not CD25 was blocked, the grade of gastritis was significantly higher than that in the CD25 blocked and non-blocked control groups (P<0.001). The grade of gastritis in the CD25-blocked H. pylori infection group was significantly higher than that in the non-blocked H. pylori infection group (P<0.05) (Fig 2B). The gastric mucosae of *H*. *pylori*-infected mice were tested for gastritis. HE staining of the gastric mucosa of the control group showed fewer inflammatory cells infiltrated into the gastric mucosa (Fig 2C, 2E and 2G, ×100). HE staining of the gastric mucosa of the *H*. *pylori* infection group showed more inflammatory cells infiltrated into the gastric mucosa than those visible in the TLR4 blocked *H*. *pylori* infection group, and milder inflammatory cell infiltration than that in the CD25 blocked *H*. *pylori* infection group (Fig 2D, 2F and 2H, ×100). (data in S3 and S4 Tables).

### Effect of *H*. *pylori* infection on Th1, Th17, and Th2

To determine the cellular immune response (as represented by Th1, Th17, and Th2 cells) with TLR4 or CD25 blockage after *H*. *pylori* infection, we measured the levels of cytokines in the gastric mucosa by ELISA.

**Fig 3:**
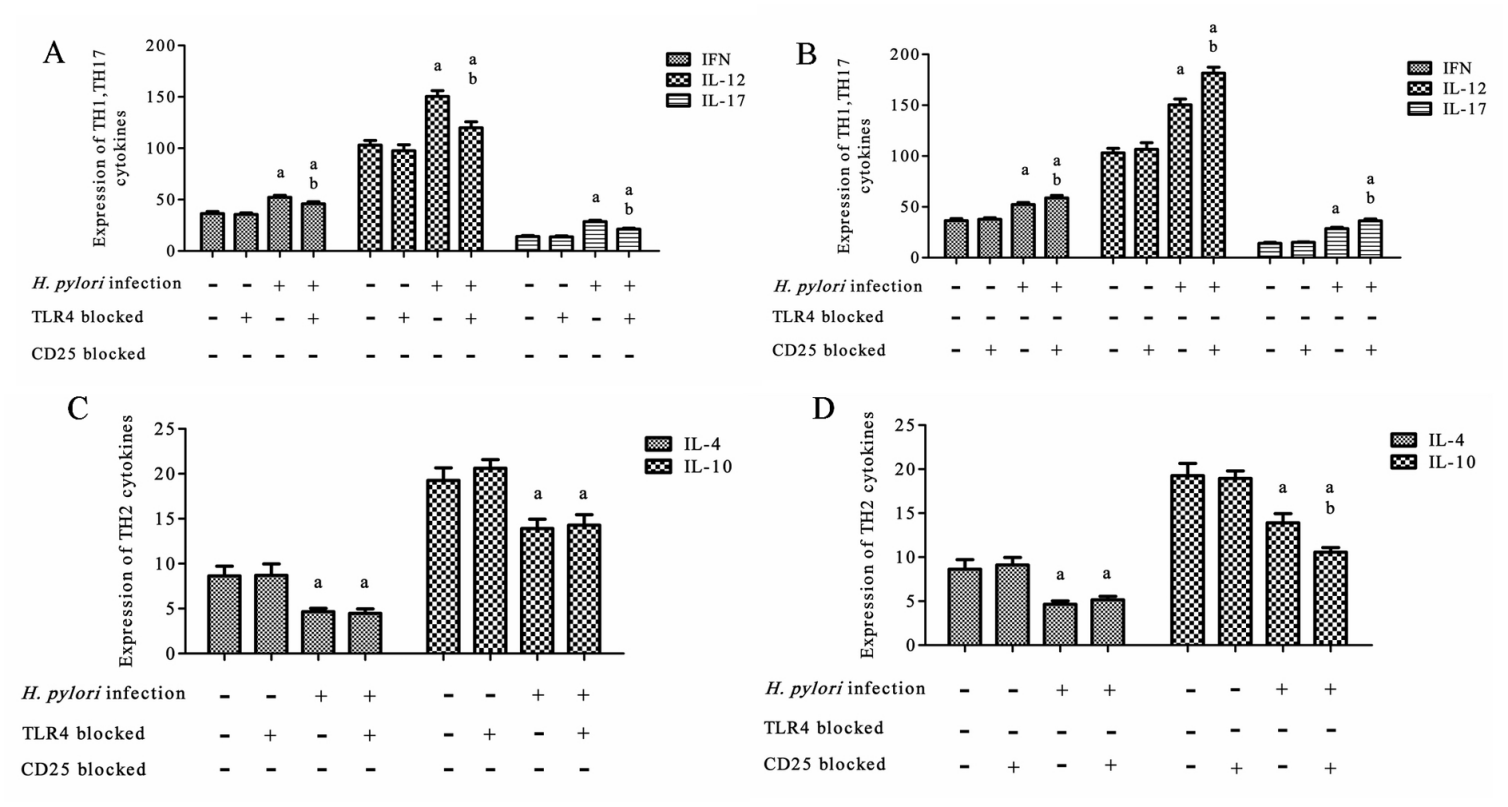
Expression of Th1, Th17, and Th2 cytokines in the gastric mucosa after *H*. *pylori* infection. (A) The expression of Th1 and Th17 with TLR4 blocked; (B) The expression of Th1 and Th17 with CD25 blocked; (C) The expression of Th2 with TLR4 blocked; (D) The expression of Th2 with CD25 blocked. ^a^*P* < 0.05–0.001 *vs*. the control or TLR4 blocked control groups; ^b^*P* < 0.001–0.05 between TLR4 blocked *H*. *pylori* and *H*. *pylori* groups (Fig 3A); ^a^*P* < 0.001 *vs*. the control and CD25 blocked control groups; ^b^*P* < 0.001–0.05 between CD25 blocked *H*. *pylori* and *H*. *pylori* groups (Fig 3B). ^a^*P* < 0.01 *vs*. control and TLR4 blocked control groups (Fig 3C); ^a^*P* < 0.01–0.001 *vs*. control and CD25 blocked control groups; ^b^*P* < 0.05 between CD25 blocked and non-blocked *H*. *pylori* groups (Fig 3D).

After *H*. *pylori* infection, the gastric mucosa of *H*. *pylori*-infected mice were tested for the expression of the Th1 and Th17 cytokines IFN, IL-12, and IL-17, and the Th2 cytokines IL-4 and IL-10. The expression of Th1 and Th17 cytokines in the non-blocked *H*. *pylori* infection group was significantly higher than that in the TLR4 blocked *H*. *pylori* infection group (*P* < 0.05–0.001) (Fig 3A), and significantly lower than that in the CD25 blocked *H*. *pylori* infection group (*P* < 0.05–0.001) (Fig 3B). The expression of Th2 cytokines was not significantly different between the TLR4 blocked and non-blocked *H*. *pylori* infection groups (*P* > 0.05) (Fig 3C), and the expression of IL-4 had no significant difference between the CD25 blocked and non-blocked *H*. *pylori* infection groups. However, the expression of IL-10 in the CD25 blocked *H*. *pylori* infection group was significantly lower than that in the non-blocked *H*. *pylori* infection group (*P* < 0.05) (Fig 3D). (data in S5–S8 Tables).

### Expression of MyD88, NF-κB p65, and Foxp3 in the gastric mucosa

To determine the expression of the TLR4 signaling pathway and of Tregs following TLR4 or CD25 blockage after *H*. *pylori* infection, we measured the levels of MyD88, NF-κB p65, and Foxp3 in the gastric mucosa by immunohistochemistry and the levels of MyD88 and NF-κB p65 in the gastric mucosa by western blotting. (data in S9–S14 Tables).

#### Immunohistochemistry

**Fig 4:**
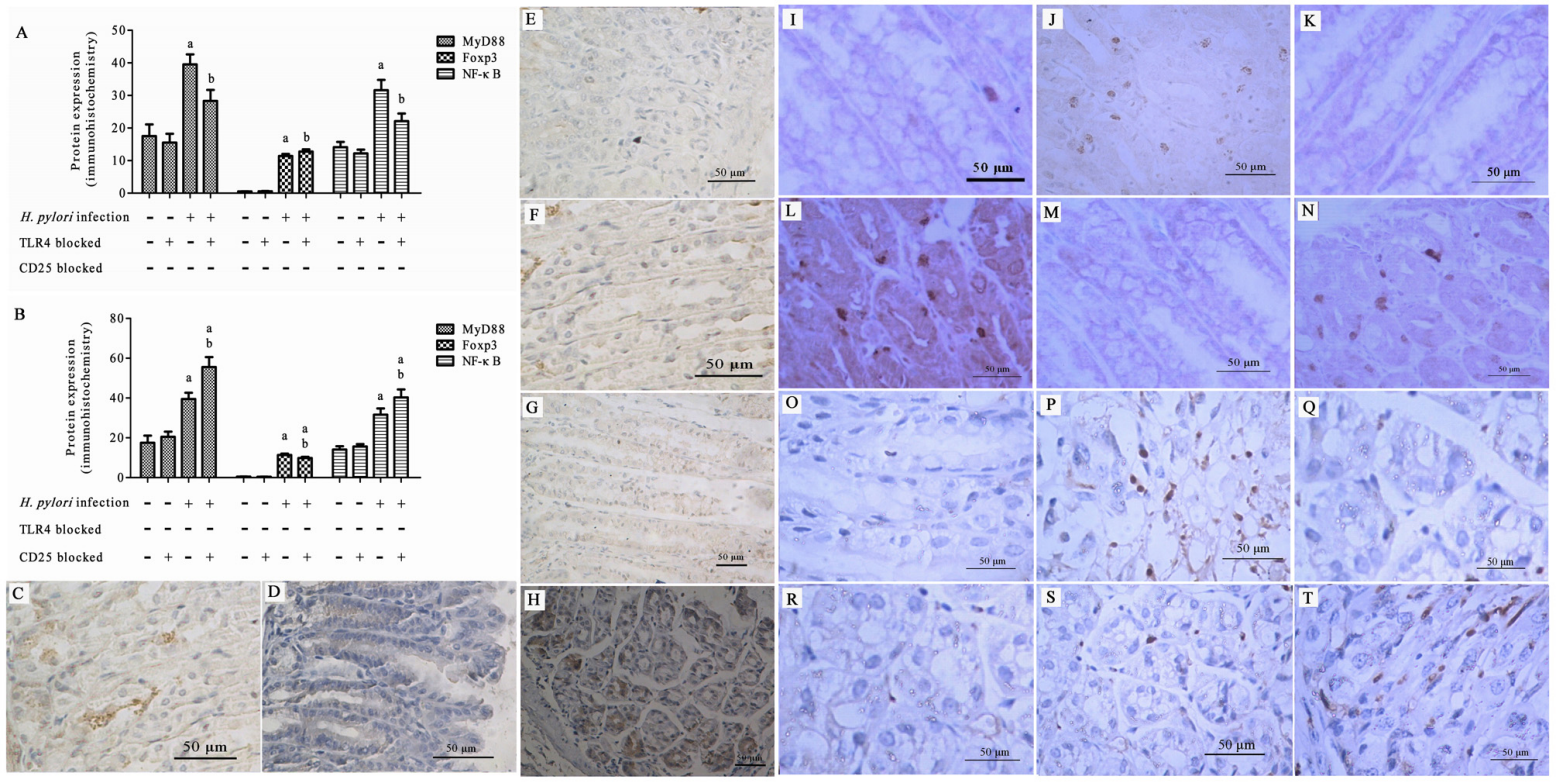
Expression of MyD88, NF-κB p65, and Foxp3 in the gastric mucosa by immunohistochemistry after *H*. *pylori* infection. Expression of MyD88, NF-κB p65, and Foxp3 with TLR4 (A) or CD25 (B) blocked; (C-T) Representative images of immunohistochemical staining. MyD88 staining in the gastric mucosa of the untreated (C, D), TLR4 blocked (E, F), or CD25 blocked (G, H) control and *H*. *pylori* infection groups; NF-κB p65 staining of the gastric mucosa of the untreated (I, J), TLR4 blocked (K, L), or CD25 blocked (M, N) control and *H*. *pylori* infection groups; Foxp3 staining of the gastric mucosa of the untreated (O, P), TLR4 blocked (Q, R), or CD25 blocked (S, T) control and *H*. *pylori* infection groups. ^a^*P* < 0.001 *vs*. control or TLR4 blocked control groups; ^b^*P* < 0.001–0.05 *vs*. control and TLR4 blocked control groups and the *H*. *pylori* group (Fig 4A). ^a^*P* < 0.001–0.01 *vs*. control and CD25 blocked control groups; ^b^*P* < 0.01–0.05 between CD25 blocked *H*. *pylori* and *H*. *pylori* groups (Fig 4B).

After *H*. *pylori* infection, the gastric mucosae of *H*. *pylori*-infected mice were tested for the expression of MyD88, NF-κB p65, and Foxp3. The expression of MyD88 and NF-κB p65 in the non-blocked *H*. *pylori* infection group was significantly higher than that in the TLR4 blocked *H*. *pylori* infection group (*P* < 0.01–0.05), and significantly lower than that in the CD25 blocked *H*. *pylori* infection group (*P* < 0.05). The expression of Foxp3 in the non-blocked *H*. *pylori* infection group was significantly lower than that in the TLR4 blocked *H*. *pylori* infection group (*P* < 0.05), and significantly higher than that in the CD25 blocked *H*. *pylori* infection group (*P* < 0.05) (Fig 4A and 4B).

MyD88 immunohistochemical staining of the gastric mucosa of the control group with or without TLR4 or CD25 blockage showed few positive cells (Fig 4C, 4E and 4G, ×200). In contrast, immunohistochemical staining of the gastric mucosa of the *H*. *pylori* infection group with or without TLR4 or CD25 blockage showed abundant positive cells (Fig 4D, 4F and 4H, ×200). MyD88 immunohistochemical staining of the gastric mucosa of the *H*. *pylori* infection group showed more positive cells than could be seen in the TLR4 blocked *H*. *pylori* infection group, and fewer positive cells than in the CD25 blocked *H*. *pylori* infection group. (Fig 4D, 4F and 4H, ×200).

NF-κB p65 immunohistochemical staining of the gastric mucosa of the control group with or without TLR4 or CD25 blockage showed few positive cells (Fig 4I, 4K and 4M, ×200), whereas immunohistochemical staining of the gastric mucosa of the *H*. *pylori* infection group with or without TLR4 or CD25 blockage showed abundant positive cells (Fig 4J, 4L and 4N, ×200). NF-κB p65 immunohistochemical staining of the gastric mucosa of the *H*. *pylori* infection group showed more positive cells than could be observed in the TLR4 blocked *H*. *pylori* infection group, and fewer positive cells than in the CD25 blocked *H*. *pylori* infection group (Fig 4J, 4L and 4N, ×200).

Foxp3 immunohistochemical staining of the gastric mucosa of the control group with or without TLR4 or CD25 blockage showed few positive cells (Fig 4O, 4Q and 4S, ×200), whereas immunohistochemical staining of the gastric mucosa of the *H*. *pylori* infection group with or without TLR4 or CD25 blockage showed abundant positive cells (Fig 4P, 4R and 4T, ×200). Foxp3 immunohistochemical staining of the gastric mucosa of the *H*. *pylori* infection group showed fewer positive cells than were observed in the TLR4 blocked *H*. *pylori* infection group, and more positive cells than in the CD25 blocked *H*. *pylori* infection group (Fig 4P, 4R and 4T, ×200).

#### Western blotting

**Fig 5:**
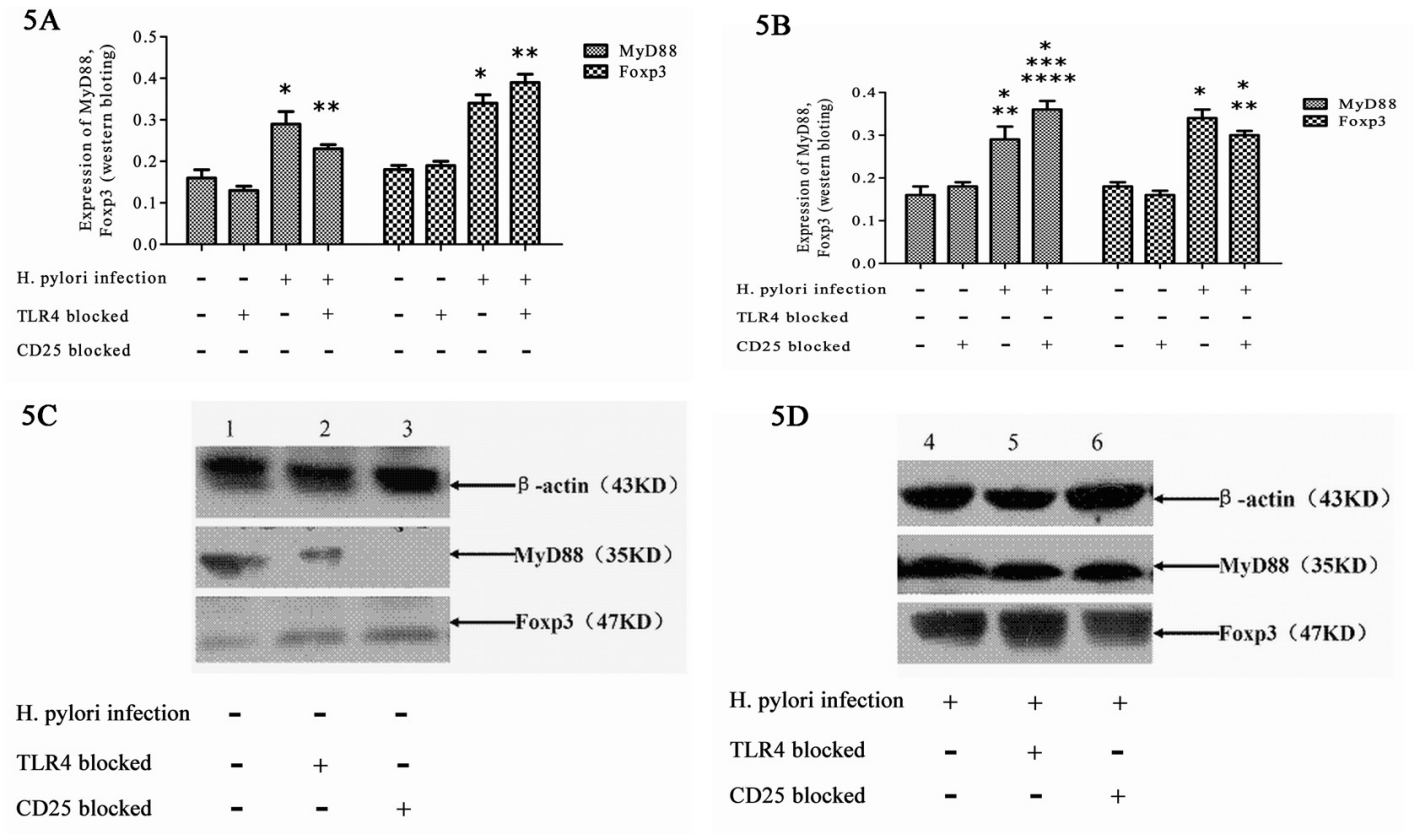
Expression of MyD88 and Foxp3 in the gastric mucosa after *H*. *pylori* infection by western blot. (A) The expression of MyD88 and Foxp3 with TLR4 blocked; (B) The expression of MyD88 and Foxp3 with CD25 blocked; (C, D) The expression of MyD88 and Foxp3 by western blotting. ^a^*P* < 0.001 *vs*. control and TLR4 blocked control groups; ^b^*P* < 0.001–0.05 *vs*. control and TLR4 blocked control groups and the *H*. *pylori* group (Fig 5A). ^a^*P* < 0.01–0.001 *vs*. control and CD25 blocked control groups; ^b^*P* < 0.05 between CD25 blocked *H*. *pylori* and *H*. *pylori* groups (Fig 5B).

After *H*. *pylori* infection, the gastric mucosae of *H*. *pylori*-infected mice were tested for the expression of MyD88 and Foxp3 by western blotting. The size of β-actin was 43 kD, the size of MyD88 was 35 kD, and the size of Foxp3 was 47 kD. MyD88 and Foxp3 western blot analysis of the gastric mucosa of the control groups with or without TLR4 or CD25 blockage showed only light bands (Fig 5C), whereas strong bands were observed for the gastric mucosa of the *H*. *pylori* infection groups with or without TLR4 or CD25 blockage (Fig 5D). MyD88 western blot analysis of the gastric mucosa of the *H*. *pylori* infection group showed a stronger band than that from the TLR4 blocked *H*. *pylori* infection group, and a lighter band than that from the CD25 blocked *H*. *pylori* infection group. Foxp3 western blot analysis of the gastric mucosa of the *H*. *pylori* infection group showed a lighter band than that from the TLR4 blocked *H*. *pylori* infection group, and a stronger band than that from the CD25 blocked *H*. *pylori* infection group (Fig 5D).

## Discussion

*H*. *pylori*, a highly adaptable gram-negative bacillus, can colonize the human gastric system and induce diseases such as gastric ulcer and carcinoma [24]. Our previous study [25] found that after infection by *H*. *pylori*, the *H*. *pylori* antigen induced CD4^+^ Th1 cells in the gastric mucosa of mice, producing special cellular immunity through antigen-presenting cells. However, this cellular immunity offered no protection against *H*. *pylori* infection in these animals, and was associated with disease severity. In addition, IL-4 and IL-10 secretion by CD4^+^ Th2 cells was suppressed, which resulted in a decrease of the levels of IgA secreted by B cells. Therefore, although powerful local and systematic humoral and cellular immunities were produced in the mucosa after *H*. *pylori* infection, this immunity response could not eliminate the bacillus, and permitted the persistence of chronic infection throughout the individual’s lifespan. That led to a constant progression of gastric and duodenum disease, including tumor occurrence. Therefore, we studied the association and interaction between the TLR4 signaling pathway and Tregs in the gastric and gut mucosa of mice infected with *H*. *pylori*, in order to illuminate the mechanism by which *H*. *pylori* escapes elimination by the host immune response mechanism.

Our previous study showed that the Th1/Th2 immune response could only be effective at eliminating bacteria or protecting the host against infection when the cells reached a specified balance [26]. Here, we identified that the responses of Th17 to manipulation of the T cell signaling pathway were similar to those of Th1. In accordance with this, an increasing number of studies have found that Th17 also plays an important role in the defense against *H*. *pylori*, which can cause gastritis and peptic ulcer due to the increased activation of Th17 and cytokine changes [27]. Munari et al. [28] investigated Th17 response in *H*. *pylori*-associated gastritis in humans, and found that there was a high level of IL-17 cytokines in the gastric mucosa of *H*. *pylori*-positive patients with chronic gastritis, whereas IL-17 was only weakly expressed in *H*. *pylori*-negative patients with chronic gastritis; in addition, IL-17 levels were found to decrease after eradication of the bacteria. These results suggest that Th17 immunity response might also influence the elimination of *H*. *pylori*.

### Role of the TLR4 signaling pathway in *H*. *pylori* colonization and inflammation

TLR has been shown to be the key protein for inflammatory signal transmission. Once activated, TLRs not only induced inflammatory response, but also built an antigen-related special adaptive immunity [29]. The TLR signaling pathway consists of a TLR extracellular domain containing leucine-rich repeat motifs and an intracellular domain containing a Toll/IL-1R receptor domain (TIR domain), myeloid differentiation marker 88 (MyD88), interleukin receptor-associated kinase (IRAK), and TNF receptor-associated factor (TRAF) 6. After a series of substance-mediated activations, nuclear factor-κB (NF-κB)-induced protein kinase (NIK) becomes activated and subsequently activates NF-κB itself. Activated NF-κB could trigger cytokine gene transcription and cause a subsequent immune and inflammation response [30]. Thus, MyD88 is a critical component of TLR signaling, and most TLRs utilize the MyD88-dependent signaling pathway, including TLR2, TLR4, TLR5, TLR7, TLR8, and TLR9 [31]. Among these molecules, TLR2, TLR4, and TLR9 all have the potential to be expressed on gastric epithelial cells. Lagunes-Servin et al. [32] examined the gastric biopsies of children and found that *H*. *pylori* infection was associated with significantly increased expression of TLRs 2, 4, 5, and 9, demonstrating that the gastric epithelia of children respond to *H*. *pylori* infection by increasing the expression of these TLRs. Sun et al. [33] further found that as the degree of gastritis was increased, *H*. *pylori* colonization was decreased, and that the Th1 response was enhanced in *H*. *pylori*-infected TLR2-deficient mice, but not in TLR4-deficient mice. Baneriee et al. [34] found that in MyD88-deficient (MyD88(-/-)) mice, Helicobacter infection resulted in the advancement to gastric dysplasia, and showed evidence of MyD88 protection in an infection-driven inflammation-associated cancer model. These studies all showed that TLR signaling pathways had a close association with *H*. *pylori* infection. In turn, our study found that blockage of TLR4 led to a reduction of the downstream molecules MyD88 and NF-κB, suppressed inflammation, and decreased the levels of Th1 and Th17 cytokines, which demonstrated that TLR4 could promote an inflammation-based immunity response. However, we identified no significant difference in the Th2 cytokines between the TLR4 blocked and non-blocked groups; this might be because the Th2 cytokines might be induced by the *H*. *pylori* vaccine to protect against *H*. *pylori*, but not be induced by *H*. *pylori* infection directly. Furthermore, blockage of TLR4 also increased the density of *H*. *pylori* colonization, although it decreased inflammation response. Our previous study [35] found that application of an *H*. *pylori* whole cell therapeutic vaccine significantly increased the expression of TLR4 in gastric mucosa and reduced *H*. *pylori* colonization. These results showed that although TLR4 had a role in *H*. *pylori* elimination, it elicited gastric mucosa damage. Therefore, TLR4 might have an important role in immune prevention and in the treatment effects of an *H*. *pylori* vaccine.

### Tregs in *H*. *pylori* colonization and inflammation

Increasing amounts of evidence has pointed to the likelihood that *H*. *pylori* might induce a Treg immune response against helper T cell immunity that could lead to persistent infection. For example, Rad et al. [36] used an anti-CD25 monoclonal antibody (PC61) to deplete the CD25+ cells of C57BL/6 mice, and found that severe gastritis developed as a consequence, with heightened cytokine expression and increased numbers of mucosal T cells, B cells, and macrophages, which led to increased gastric inflammation and reduced bacterial colonization. We also found that blockade of CD25 not only reduced levels of the downstream molecule Foxp3, but also exacerbated the inflammation associated with *H*. *pylori* infection and increased the levels of Th1 and Th17 cytokines, which demonstrated that Tregs suppressed the inflammatory immune response. Similar to our findings for TLR4, the Th2 cytokine IL-4 also showed no difference between the CD25-blocked and non-blocked groups. Additionally, increased numbers of Tregs also accompanied the increased density of *H*. *pylori* colonization, as previously observed. Similarly, our previous study [35] found that an *H*. *pylori* whole-cell therapeutic vaccine significantly decreased the expression of Foxp3 in gastric mucosa, which reduced *H*. *pylori* colonization. Together, these results demonstrated that Tregs might also take part in the mechanism by which *H*. *pylori* escapes host elimination. However, Rad et al. did not study the change in TLR4 expression with the change in Treg numbers. Therefore, our study provides new information on the interaction between the TLR signaling pathway and Tregs.

### Interaction between the TLR signaling pathway and Tregs

The TLR signaling pathway and Tregs both exhibited a close association with *H*. *pylori* colonization and inflammation after infection. Sun et al. [33] also found that when TLR2 was knocked out in mice, the expression of Foxp3 was suppressed; however, they did not study the change of TLR signaling after Treg blockage. Furthermore, we found that blockage of TLR4 increased the expression of the Treg-specific marker Foxp3 in gastric mucosa and exacerbated the numbers of CD4^+^CD25^+^Foxp3^+^ Tregs, and that blockage of CD25 up-regulated MyD88 expression and promoted NF-κB activation, exacerbating the effects of the TLR4 signaling pathway. These findings suggested that the TLR4 signaling pathway and Tregs had a negative correlation. Once TLR4 was blocked, *H*. *pylori* colonization would increase and inflammation would have been reduced. On the other hand, CD25 blockage would reduce *H*. *pylori* colonization and increase the inflammation. Additionally, TLR4 and Tregs also interacted with each other; our results suggested that this interaction might result in a more effective elimination of *H*. *pylori* during immune regulation. This scenario also indicates a new direction for developing a treatment schedule against *H*. *pylori*.

## Conclusions

Our results suggest that blocking the TLR4 signaling pathway could down-regulate MyD88 expression, reduce NF-κB activation, and increase the numbers of CD4^+^CD25^+^Foxp3^+^ Tregs. This in turn might depress the Th1 and Th17 immune response, which could exacerbate *H*. *pylori* colonization density and reduce the degree of inflammation in the gastric mucosa of mice infected with *H*. *pylori*. In contrast, blocking CD25 could up-regulate MyD88 expression, promote NF-κB activation and decrease the numbers of CD4^+^CD25^+^Foxp3^+^ Tregs, which would promote the Th1 and depress the Th2 immune response, in turn reducing *H*. *pylori* colonization density and exacerbating the degree of inflammation in the gastric mucosa of mice infected with *H*. *pylori*. Therefore, the interaction between the TLR signaling pathway and Tregs might be an important factor in reducing *H*. *pylori* colonization and suppressing the inflammation response of mice infected with *H*. *pylori*. This mechanism might provide a new strategy for designing effective preventive and therapeutic treatment regimens against initial and persistent *H*. *pylori* infection.

## Supporting Information

S1 Table *H*. *pylori* colonization score in the gastric mucosa with TLR4 blocked after infection. (DOC)

S2 Table *H*. *pylori* colonization score in the gastric mucosa with CD25 blocked after infection. (DOC)

S3 Table Grade of gastritis with TLR4 blocked after infection. (DOC)

S4 Table Grade of gastritis with CD25 blocked after infection. (DOC)

S5 Table Expression of Th1, Th17 cytokines in the gastric mucosa with TLR4 blocked after infection. (DOC)

S6 Table Expression of Th1, Th17 cytokines in the gastric mucosa with CD25 blocked after infection. (DOC)

S7 Table Expression of Th2 cytokines in the gastric mucosa with TLR4 blocked after infection. (DOC)

S8 Table Expression of Th2 cytokines in the gastric mucosa with CD25 blocked after infection. (DOC)

S9 Table Expression of MyD88 in the gastric mucosa with TLR4 blocked after infection. (DOC)

S10 Table Expression of Foxp3 in the gastric mucosa with TLR4 blocked after infection. (DOC)

S11 Table Expression of NF-κB p65 in the gastric mucosa with TLR4 blocked after infection by immunohistochemistry. (DOC)

S12 Table Expression of MyD88 in the gastric mucosa with CD25 blocked after infection. (DOC)

S13 Table Expression of Foxp3 in the gastric mucosa with CD25 blocked after infection. (DOC)

S14 Table Expression of NF-κB p65 in the gastric mucosa with CD25 blocked after infection by immunohistochemistry. (DOC)
