# Supplementary material for: Association of TLR4 and Treg in Helicobacter pylori Colonization and Inflammation in Mice
Source: PLoS One. 2016 Feb 22;11(2):e0149629. doi: 10.1371/journal.pone.0149629 (PMC4762684; doi:10.1371/journal.pone.0149629)
Supplement: S1 Table — (DOC) [file pone.0149629.s001.doc]

**S1 Table. *H. pylori* colonization score in the gastric mucosa with TLR4 blocked after infection.**

| Groups | N | *H. pylori* colonization score | | | | |
| --- | --- | --- | --- | --- | --- | --- |
|  |  | 0 | 1 | 2 | 3 | 4 |
| ①Control group | 10 | 10 | 0 | 0 | 0 | 0 |
| ②TLR4 blocked control group | 10 | 10 | 0 | 0 | 0 | 0 |
| ③*H. pylori* group ^b^ | 10 | 0 | 2 | 5 | 2 | 1 |
| ④TLR4 blocked *H. pylori* group ^a^ | 9 | 0 | 0 | 3 | 5 | 1 |

^a^*P* < 0.001vs ①②groups; ^b^*P*< 0.05 vs ④ group.
