# Supplementary material for: Association of TLR4 and Treg in Helicobacter pylori Colonization and Inflammation in Mice
Source: PLoS One. 2016 Feb 22;11(2):e0149629. doi: 10.1371/journal.pone.0149629 (PMC4762684; doi:10.1371/journal.pone.0149629)
Supplement: S2 Table — (DOC) [file pone.0149629.s002.doc]

**S2 Table. *H. pylori* colonization score in the gastric mucosa with CD25 blocked after infection.**

| Groups | N | *H. pylori* colonization score | | | | |
| --- | --- | --- | --- | --- | --- | --- |
|  |  | 0 | 1 | 2 | 3 | 4 |
| ①Control group | 10 | 10 | 0 | 0 | 0 | 0 |
| ②CD25 blocked control group | 10 | 10 | 0 | 0 | 0 | 0 |
| ③*H. pylori* group ^a、b^ | 10 | 0 | 2 | 5 | 2 | 1 |
| ④CD25 blocked *H. pylori* group ^a^ | 10 | 0 | 5 | 4 | 1 | 0 |

^a^*P* < 0.001vs ①②groups; ^b^*P*< 0.05 vs ④ group.
