# Supplementary material for: Association of TLR4 and Treg in Helicobacter pylori Colonization and Inflammation in Mice
Source: PLoS One. 2016 Feb 22;11(2):e0149629. doi: 10.1371/journal.pone.0149629 (PMC4762684; doi:10.1371/journal.pone.0149629)
Supplement: S3 Table — (DOC) [file pone.0149629.s003.doc]

**S3 Table. Grade of gastritis with TLR4 blocked after infection.**

| Groups | N | The grade of gastritis | | | |
| --- | --- | --- | --- | --- | --- |
|  |  | 0 | 1 | 2 | 3 |
| ①Control group | 10 | 8 | 2 | 0 | 0 |
| ②TLR4 blocked control group | 10 | 6 | 4 | 0 | 0 |
| ③*H. pylori* group ^a^ | 10 | 0 | 4 | 4 | 2 |
| ④TLR4 blocked *H. pylori* group ^b、c^ | 10 | 2 | 4 | 4 | 0 |

^a^*P* < 0.001vs ①②groups; ^b^ *P* < 0.01vs ①②groups; ^c^*P*< 0.05 vs ③ group.
