# Supplementary material for: Association of TLR4 and Treg in Helicobacter pylori Colonization and Inflammation in Mice
Source: PLoS One. 2016 Feb 22;11(2):e0149629. doi: 10.1371/journal.pone.0149629 (PMC4762684; doi:10.1371/journal.pone.0149629)
Supplement: S4 Table — (DOC) [file pone.0149629.s004.doc]

**S4 Table. Grade of gastritis with CD25 blocked after infection.**

| Groups | N | The grade of gastritis | | | |
| --- | --- | --- | --- | --- | --- |
|  |  | 0 | 1 | 2 | 3 |
| ①Control group | 10 | 8 | 2 | 0 | 0 |
| ②CD25 blocked control group | 10 | 7 | 3 | 0 | 0 |
| ③*H. pylori* group ^a、b^ | 10 | 0 | 4 | 4 | 2 |
| ④CD25 blocked *H. pylori* group^、a^ | 10 | 0 | 1 | 4 | 5 |

^a^*P* < 0.001vs ①②groups; ^b^ *P*< 0.05 vs ④ group.
