# Supplementary material for: Association of TLR4 and Treg in Helicobacter pylori Colonization and Inflammation in Mice
Source: PLoS One. 2016 Feb 22;11(2):e0149629. doi: 10.1371/journal.pone.0149629 (PMC4762684; doi:10.1371/journal.pone.0149629)
Supplement: S5 Table — (DOC) [file pone.0149629.s005.doc]

**S5 Table. Expression of Th1, Th17 cytokines in the gastric mucosa with TLR4 blocked after infection.**

| Groups | N | IFN | IL-12 | IL-17 |
| --- | --- | --- | --- | --- |
| ①Control group | 6 | 36.41±1.95 | 103.03±4.58 | 14.09±0.95 |
| ②TLR4 blocked control group | 6 | 35.53±1.60 | 97.56±5.85 | 13.67±1.11 |
| ③*H. pylori* group | 6 | 52.29±1.94^a^ | 150.42±5.59^a^ | 28.51±1.36^a^ |
| ④TLR4 blocked *H. pylori* group | 6 | 45.85±2.09^b 、c^ | 119.87±5.87^b、d^ | 21.32±0.89^e^ |

^a^*P* < 0.001vs ①②groups; ^b^ *P*< 0.05 vs ③ group; ^c^*P* < 0.01vs ①②groups; ^d^*P* < 0.05vs ①②groups; ^e^*P* < 0.001vs ①②③groups.
