# Supplementary material for: Association of TLR4 and Treg in Helicobacter pylori Colonization and Inflammation in Mice
Source: PLoS One. 2016 Feb 22;11(2):e0149629. doi: 10.1371/journal.pone.0149629 (PMC4762684; doi:10.1371/journal.pone.0149629)
Supplement: S6 Table — (DOC) [file pone.0149629.s006.doc]

**S6 Table. Expression of Th1, Th17 cytokines in the gastric mucosa with CD25 blocked after infection.**

| Groups | N | IFN | IL-12 | IL-17 |
| --- | --- | --- | --- | --- |
| ①Control group | 6 | 36.41±1.95 | 103.03±4.58 | 14.09±0.95 |
| ②CD25 blocked control group | 6 | 37.76±1.47 | 106.59±6.41 | 15.02±0.55 |
| ③*H. pylori* group | 6 | 52.29±1.94^a^ | 150.42±5.59^a^ | 28.51±1.36^a^ |
| ④CD25 blocked *H. pylori* group | 6 | 58.75±2.42^a、b^ | 181.51±5.95^a、b^ | 36.25±1.90^c^ |

^a^*P* < 0.001vs ①②groups; ^b^ *P*< 0.05 vs ③ group; ^c^*P* < 0.001vs ①②③groups.
