# Supplementary material for: Association of TLR4 and Treg in Helicobacter pylori Colonization and Inflammation in Mice
Source: PLoS One. 2016 Feb 22;11(2):e0149629. doi: 10.1371/journal.pone.0149629 (PMC4762684; doi:10.1371/journal.pone.0149629)
Supplement: S7 Table — (DOC) [file pone.0149629.s007.doc]

**S7 Table. Expression of Th2 cytokines in the gastric mucosa with TLR4 blocked after infection.**

| Groups | N | IL-4 | IL-10 |
| --- | --- | --- | --- |
| ①Control group | 6 | 8.64±1.08 | 19.25±1.41 |
| ②TLR4 blocked control group | 6 | 8.70±1.27 | 20.61±0.96 |
| ③*H. pylori* group | 6 | 4.65±0.39^a^ | 13.90±1.05^a^ |
| ④TLR4 blocked *H. pylori* group | 6 | 4.46±0.51^a^ | 14.29±1.16^a^ |

^a^*P* < 0.01vs ①②groups
