# Supplementary material for: Association of TLR4 and Treg in Helicobacter pylori Colonization and Inflammation in Mice
Source: PLoS One. 2016 Feb 22;11(2):e0149629. doi: 10.1371/journal.pone.0149629 (PMC4762684; doi:10.1371/journal.pone.0149629)
Supplement: S8 Table — (DOC) [file pone.0149629.s008.doc]

**S8 Table. Expression of Th2 cytokines in the gastric mucosa with CD25 blocked after infection.**

| Groups | N | IL-4 | IL-10 |
| --- | --- | --- | --- |
| ①Control group | 6 | 8.64±1.08 | 19.25±1.41 |
| ②CD25 blocked control group | 6 | 9.12±0.84 | 18.95±0.86 |
| ③*H. pylori* group | 6 | 4.65±0.39^a^ | 13.90±1.05^a^ |
| ④CD25 blocked *H. pylori* group | 6 | 5.15±0.41^a^ | 10.58±0.51^b、c^ |

^a^*P* < 0.01vs ①②groups; ^b^ *P* < 0.001vs ①②groups; ^c^ *P*< 0.05 vs ③ group.
