# Supplementary material for: Association of TLR4 and Treg in Helicobacter pylori Colonization and Inflammation in Mice
Source: PLoS One. 2016 Feb 22;11(2):e0149629. doi: 10.1371/journal.pone.0149629 (PMC4762684; doi:10.1371/journal.pone.0149629)
Supplement: S9 Table — (DOC) [file pone.0149629.s009.doc]

**S9 Table. Expression of MyD88 in the gastric mucosa with TLR4 blocked after infection.**

| Groups | N | immunohistochemistry | Western blot |
| --- | --- | --- | --- |
| ①Control group | 10 | 17.50±3.59 | 0.16±0.02 |
| ②TLR4 blocked control group | 10 | 15.50±2.73 | 0.13±0.01 |
| ③*H. pylori* group | 10 | 39.50±3.11 ^a^ | 0.29±0.03^a^ |
| ④TLR4 blocked *H. pylori* group | 10 | 28.30±3.37 ^b、c^ | 0.23±0.01^b、c^ |

^a^*P* < 0.001vs ①②groups; ^b^ *P* < 0.01vs ②group; ^c^ *P*< 0.05 vs ①③ groups
