# Supplementary material for: Association of TLR4 and Treg in Helicobacter pylori Colonization and Inflammation in Mice
Source: PLoS One. 2016 Feb 22;11(2):e0149629. doi: 10.1371/journal.pone.0149629 (PMC4762684; doi:10.1371/journal.pone.0149629)
Supplement: S10 Table — (DOC) [file pone.0149629.s010.doc]

**S10 Table. Expression of Foxp3 in the gastric mucosa with TLR4 blocked after infection.**

| Groups | N | immunohistochemistry | Western blot |
| --- | --- | --- | --- |
| ①Control group | 10 | 0.50±0.07 | 0.18±0.01 |
| ②TLR4 blocked control group | 10 | 0.56±0.07 | 0.19±0.01 |
| ③*H. pylori* group | 10 | 11.38±0.65 ^a^ | 0.34±0.02^a^ |
| ④TLR4 blocked *H. pylori* group | 10 | 12.76±0.69 ^a、b^ | 0.39±0.02^a、b^ |

^a^*P* < 0.001vs ①②groups; ^b^ *P*< 0.05 vs ③ group.
