# Supplementary material for: Association of TLR4 and Treg in Helicobacter pylori Colonization and Inflammation in Mice
Source: PLoS One. 2016 Feb 22;11(2):e0149629. doi: 10.1371/journal.pone.0149629 (PMC4762684; doi:10.1371/journal.pone.0149629)
Supplement: S11 Table — (DOC) [file pone.0149629.s011.doc]

**S11 Table. Expression of NF-κB p65 in the gastric mucosa with TLR4 blocked after infection by immunohistochemistry.**

| Groups | N | NF-κB p65 |
| --- | --- | --- |
| ①Control group | 10 | 14.10±1.64 |
| ②TLR4 blocked control group | 10 | 12.20±1.14 |
| ③*H. pylori* group | 10 | 31.60±3.18 ^a^ |
| ④TLR4 blocked *H. pylori* group | 10 | 22.10±2.33 ^b、c^ |

^a^*P* < 0.001vs ①②groups; ^b^ *P* < 0.01vs ②③groups; ^c^ *P*< 0.05 vs ①group.
