# Supplementary material for: Association of TLR4 and Treg in Helicobacter pylori Colonization and Inflammation in Mice
Source: PLoS One. 2016 Feb 22;11(2):e0149629. doi: 10.1371/journal.pone.0149629 (PMC4762684; doi:10.1371/journal.pone.0149629)
Supplement: S12 Table — (DOC) [file pone.0149629.s012.doc]

**S12 Table. Expression of MyD88 in the gastric mucosa with CD25 blocked after infection.**

| Groups | N | immunohistochemistry | Western blot |
| --- | --- | --- | --- |
| ①Control group | 10 | 17.50±3.59 | 0.16±0.02 |
| ②CD25 blocked control group | 10 | 20.50±2.63 | 0.18±0.01 |
| ③*H. pylori* group | 10 | 39.50±3.11 ^a、b^ | 0.29±0.03^a、b^ |
| ④CD25 blocked *H. pylori* group | 10 | 55.60±4.97 ^a、c、d^ | 0.36±0.02^a、d、e^ |

^a^*P* < 0.001vs ①group; ^b^ *P*< 0.01 vs ②group; ^c^*P* < 0.01vs ③group; ^d^*P* < 0.001vs ②group; ^e^*P* < 0.05vs③group.
