# Supplementary material for: Association of TLR4 and Treg in Helicobacter pylori Colonization and Inflammation in Mice
Source: PLoS One. 2016 Feb 22;11(2):e0149629. doi: 10.1371/journal.pone.0149629 (PMC4762684; doi:10.1371/journal.pone.0149629)
Supplement: S13 Table — (DOC) [file pone.0149629.s013.doc]

**S13 Table. Expression of Foxp3 in the gastric mucosa with CD25 blocked after infection.**

| Groups | N | immunohistochemistry | Western blot |
| --- | --- | --- | --- |
| ①Control group | 10 | 0.50±0.07 | 0.18±0.01 |
| ②CD25 blocked control group | 10 | 0.46±0.04 | 0.16±0.01 |
| ③*H. pylori* group | 10 | 11.38±0.65 ^a^ | 0.34±0.02^a^ |
| ④CD25 blocked *H. pylori* group | 10 | 9.82±0.59 ^a、b^ | 0.30±0.01^a、b^ |

^a^*P* < 0.001vs ①②groups; ^b^ *P* < 0.05vs ③ group.
