# Supplementary material for: Association of TLR4 and Treg in Helicobacter pylori Colonization and Inflammation in Mice
Source: PLoS One. 2016 Feb 22;11(2):e0149629. doi: 10.1371/journal.pone.0149629 (PMC4762684; doi:10.1371/journal.pone.0149629)
Supplement: S14 Table — (DOC) [file pone.0149629.s014.doc]

**S14 Table. Expression of NF-κB p65 in the gastric mucosa with CD25 blocked after infection by immunohistochemistry.**

| Groups | N | NF-κB p65 |
| --- | --- | --- |
| ①Control group | 10 | 14.10±1.64 |
| ②CD25 blocked control group | 10 | 15.60±1.22 |
| ③*H. pylori* group | 10 | 31.60±3.18 ^a^ |
| ④CD25 blocked *H. pylori* group抗 | 10 | 40.30±4.03 ^a、b^ |

^a^*P* < 0.001vs ①②groups; ^b^ *P* < 0.05vs ③ group.
